# Finish with a sprint: Evidence for time‐selected last leg of migration in a long‐distance migratory songbird

**DOI:** 10.1002/ece3.4206

**Published:** 2018-06-21

**Authors:** Martins Briedis, Steffen Hahn, Miloš Krist, Peter Adamík

**Affiliations:** ^1^ Department of Bird Migration Swiss Ornithological Institute Sempach Switzerland; ^2^ Department of Zoology Palacký University Olomouc Czech Republic; ^3^ Museum of Natural History Olomouc Czech Republic

**Keywords:** geolocator, long‐distance migrant, migration speed, migration‐breeding transition, optimal migration, sprint migration

## Abstract

Under time‐selected migration, birds should choose a strategy for outcompeting rivals over securing access to prime resources at the final destination. Thus, migration can be viewed as a race among individuals where winners are arriving first when conditions are suitable. The sprint migration hypothesis predicts that individuals shift from maximum sustained speed to a final burst of sprint to shorten the transition from migration to breeding (Alerstam, 2006). In this study, we test the hypothesis of a final sprint migration in a long‐distance Afro‐Palearctic migrant, the collared flycatcher *Ficedula albicollis*, during autumn and spring, and compare migration strategies between the seasons. In both seasons, collared flycatchers evidently exhibited sprint migration by increasing their overall speed over the last leg of migration after the Sahara crossing. This phenomenon was more pronounced in spring, contributing to overall faster spring migration and possibly highlighting higher importance for early arrival at the breeding grounds. In both seasons and particularly in spring, late departing individuals flew at a faster rate, partially being able to catch up with their early departing conspecifics. Differential fueling strategies may play an important role in determining migration speed, especially during the early stages of the migration, and might explain the observed differences in migration speeds between late and early departing individuals. Our findings suggest competition for early arrival at the breeding and at the nonbreeding destinations alike. Sprint migration might be an appropriate strategy to gain advantage over conspecifics and settle in prime territories as well as to cope with the increasingly earlier springs at high latitudes.

## 1 INTRODUCTION

The ecological background for why birds migrate differs seasonally (Newton, 2008). In autumn, the drive for migration comes from a self‐preservation viewpoint as birds try to increase their chances of survival by escaping the forthcoming unfavorable conditions at their breeding sites. In spring, however, birds urge to return to the breeding sites to exploit the seasonal increase in food availability for reproduction. Because of these season‐specific causes for migration and the variable life history events following the migration, birds may adopt different migration strategies in each of the seasons. The optimal migration theory (Alerstam, 2011) states that birds on the move should optimize their energy expenditure (transport costs)—the energy minimization strategy—and they should maximize the total migration speed—the time‐minimization strategy (Hedenström, 2008). However, the two strategies may be seen as two endpoints along a continuum and birds may adopt intermediate behaviors depending on the relative importance of energy‐ and time‐selection pressure (Alerstam & Lindström, 1990).

Passerines are typical income breeders (Langin, Norris, Kyser, Marra, & Ratcliffe, 2006) and arrive at the breeding grounds early and use the local resources for reproduction. Earlier arriving individuals, thus, outcompete their rivals for access to important assets, like prime breeding territories (Kokko, 1999). Because early arrival time is of high importance for reproductive success, we may expect the birds to optimize time, rather than energy during the spring migration, that is, to adopt the time‐minimization strategy (Alerstam & Lindström, 1990). While advantages of early arrival are evident in spring, the benefits for early arrival at the nonbreeding grounds are less obvious (Gill et al., 2001). If competition for resources, like prime molting sites, at the nonbreeding areas is fierce, we may expect similar migration strategies in respect to timely arrival in both seasons. If arrival time at the nonbreeding sites is of low importance, birds may rather choose to optimize their energy expenditure during the autumn migration, that is, to adopt the energy minimization strategy, and thus, migrate at an overall slower speed as compared to spring (Alerstam & Lindström, 1990).

While theoretical background of the arrival game is strong (Kokko, 1999), we still lack good empirical evidence of the mechanisms how individuals secure early (spring and possibly autumn) arrival to outcompete their rivals. Early arrival could be induced by departing for migration before the competitors and/or by migrating at a faster speed. Earlier tracking studies have shown that the overall migration duration in passerines is shorter and speed is higher in spring than in autumn (McKinnon, Fraser, & Stutchbury, 2013; Nilsson, Klaassen, & Alerstam, 2013; Schmaljohann, 2018). Faster migration can be achieved by flying at higher speeds or by shortening the duration of stopovers. Radar studies revealed that nocturnal migrants fly on average at 12%–16% higher speed in spring compared to autumn (Karlsson, Nilsson, Bäckman, & Alerstam, 2012; Nilsson, Bäckman, & Alerstam, 2014). However, such increase in flight speed contributes relatively little to increasing the overall migration speed, as the vast majority of time during migration is spent on stopovers, rather than actually flying (Hedenstrom & Alerstam, 1997). Therefore, the faster spring migration is more likely to originate from a reduction in stopover time. One way or another, the overall faster spring migration exposes that the importance for early arrival is essentially different in autumn and spring.

How and where migrants adjust their migration speed along the route is still not known. Birds may achieve higher overall speed by increasing the migration speed over the entire migratory journey or increasing the speed of a particular leg of migration. Alerstam (2006) has proposed the “sprint migration” hypothesis: Migratory birds may adopt a variable strategy with optimizing energy expenditure through the early part of the journey (i.e., energy minimization) followed by a period with increased migration speed to complete the migration (i.e., time‐minimization strategy). However, we lack compelling evidence that migratory birds indeed increase the migration speed when approaching their destinations.

**Figure 1:**
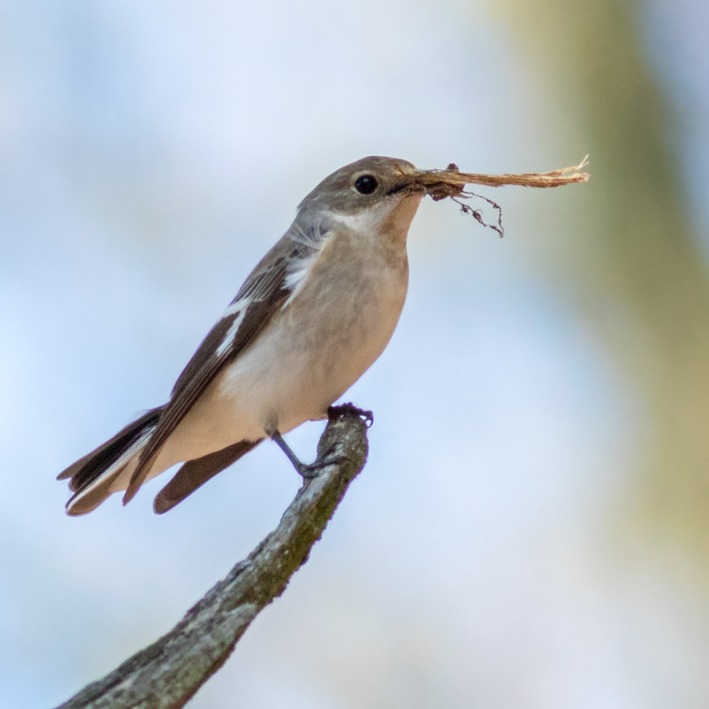
Collared flycatcher female. © Martins Briedis

In this study, we measure migration speed over different legs of the migratory journey to disentangle the strategies adopted by birds during autumn and spring migration. We use collared flycatcher *Ficedula albicollis* (Figure 1), a small long‐distance passerine migrant breeding in Europe and overwintering in south‐central Africa (Briedis, Hahn et al., 2018), as our model species. The Afro‐Palearctic bird migration system comprises of temperate and tropical–subtropical zones which are separated by a large ecological barrier—the Sahara Desert. Recent evidence from tracking studies shows rapid desert crossing by small passerines (Adamík et al., 2016; Ouwehand & Both, 2016; Xenophontos, Blackburn, & Cresswell, 2017), including the collared flycatcher, with the main stopovers typically found before the barrier crossing (Briedis, Beran, Hahn, & Adamík, 2016; Risely, Blackburn, & Cresswell, 2015). Thus, comparing migration speeds and durations prior and after the desert crossing allows for insights into migratory strategies over different legs of the migration journey. We hypothesize that (a) early arrival at the destination is of high importance in both seasons and to attain this, birds adopt the sprint migration strategy (Alerstam, 2006). Thus, we predict that migration speed will be faster for the last migration leg after the Sahara crossing in both autumn and spring. We also hypothesize (*b*) that the pressure for early arrival is larger in spring than in autumn (Kokko, 1999). Hence, we predict that the overall speed of migration will be higher and birds will advance noticeably more through the last leg of migration during spring compared to autumn. Lastly, we hypothesize that (c) sprint migration is used to outcompete rivals for early arrival at either destination. Therefore, we predict that the arrival date will highly depend on migration speed through the last leg of the migration.

## 2 METHODS

### 2.1 Study site and geolocators

We studied migration of collared flycatchers breeding at two nearby sites in the Czech Republic (Dlouhá Loučka: 49°50′N, 17°13′E and Velký Kosíř: 49°32′N, 17°04′E, distance between the sites = 30 km). Each site hosts a nest box population with ~100 breeding pairs which are monitored throughout the breeding season. In 2013 and 2014, we deployed 69 (33 males and 36 females) and 165 (157 males and 8 females) geolocators (model GDL2.0 with 7 mm light stalk, Swiss Ornithological Institute) on adult birds at Dlouhá Loučka and Velký Kosíř, respectively. Devices were attached on birds’ backs using leg‐loop silicone harnesses and each device including the harness weighted ~0.6 g (<5% of the body mass of the birds). At Dlouhá Loučka, we deployed all geolocators on breeding adults during the late nestling phase, while at Velký Kosíř, we equipped the birds with the devices upon their arrival at the site before the onset of breeding (*n* = 139) and during the breeding season (*n* = 26). Only 51 of the 139 males equipped with geolocators upon arrival at Velký Kosíř were later observed breeding in the nest boxes that season.

Next year after the deployment, we retrieved 28 geolocators (40.6%, 14 males, 14 females; +1 male 2 years later = 42% total recovery rate) at Dlouhá Loučka and 29 geolocators (17.6%, all males; +1 male 2 years later = 18.2% total recovery rate) at Velký Kosíř. The relatively low recovery rate at Velký Kosíř may at least partially be explained by the deployment of the devices on nonbreeding birds upon their spring arrival as the recovery rate was 43.3% when considering only males that were observed breeding in the nestboxes in 2014. At Dlouhá Loučka, geolocators had no noticeable effect on individual apparent survival compared to the control ringed‐only birds (return rate of control individuals = 34.9%, χ^2^ = 0.30, *p* = 0.58). Unfortunately, we lack similar data of control birds from site Velký Kosíř as all the captured males were tagged.

### 2.2 Geolocator data analyses

We defined sunrise and sunset times of the recorded light data using “GeoLocator” software (Swiss Ornithological Institute) and set the light level threshold of 1 unit from the arbitrary scale of 61 to define transition times. Further, we analyzed geolocation data using the R‐package “GeoLight” v 2.0.0 following the standard procedure (Lisovski & Hahn, 2012) outlined in Briedis, Hahn et al. (2018). We used a minimal stationary period of 3 days and probability of change (*q*‐value) 0.85 to determine stationary and movement periods with the “changeLight” function. Beginning and end of the migration periods were validated by visually inspecting changes in longitude over time as birds moved along the east–west axis. Further, geographic coordinates of individual nonbreeding sites were calculated using sun elevation angles derived from Hill‐Ekstrom calibration, while the birds were at their nonbreeding residency sites (range: +8.6 to −0.7 for different individuals). Due to the high probability of errors when determining stationary periods of short duration, we did not define any stopovers during migration. Such data would be of high risk to reflect data quality (which differed substantially among individuals as can be implied from the broad range of sun elevation angles stated above) rather than actual stopover times and sites of the flycatchers. In addition, we determined the date of Sahara crossing using prolonged periods of uninterrupted high light intensities as birds extend their nocturnal flights into the day when crossing the barrier (Adamík et al., 2016).

### 2.3 Statistical analyses

Migration period is defined as a period starting from predeparture fueling until arrival at the final destination (Alerstam, 2003). Current remote tracking techniques do not allow for quantifying the stationary fueling period before departure. Thus, we excluded this period from our estimations of migration speed, but we examine the possible effects of the predeparture fueling period for migration speeds in the discussion. Therefore, the migration speed here is defined in a conservative way as the rate of movement from the departure until arrival, including stopovers.

We excluded one outlaying individual of extraordinary long autumn migration (logger ID: 11PE, 101 days, mean = 61.1 ± 10.0 (*SD*) days) from further analyses. We used general linear models to identify whether there was an effect of study year (and accordingly study site), birds’ age (second calendar year or older), and sex on the migration speeds in autumn and spring. Analyses revealed that neither of the factors were significant predictors for migration speeds in neither of the seasons (Table 1). Thus, we pooled the data across study years, study sites, sexes, and age classes (age 5 and 6, EURING code). We selected the southern and northern edges of the Sahara Desert as the start and end points of specific migration segments generating two main migration legs for each season: (a) first leg—from departure until completion of the Sahara crossing; (b) last leg—from completion of the Sahara crossing until arrival. We included the period of Sahara crossing in the first leg of migration as barrier crossing is typically preceded by prolonged stopovers for fueling (Bayly, Atkinson, & Rumsey, 2012; Briedis, Beran et al., 2016; Risely et al., 2015; Schaub & Jenni, 2000), followed by a rapid desert crossing previously shown in collared flycatchers (Adamík et al., 2016). The duration and migration speed of the Sahara crossing of our tracked birds was 2.5 ± 0.4 days (*SD*), 828 ± 116 km/day in autumn, and 3.0 ± 0.7 days, 702 ± 154 km/day in spring. We estimated the total migration distance as a great‐circle (orthodromic) distance between the breeding site and the median location of the estimated nonbreeding site of each individual. As a result, the migration segments over Europe and over the Sahara Desert were estimated each at 2,000 km long, while the segment in sub‐Saharan Africa was ca. 3,000 km long. Please note that as we excluded the predeparture fueling period from calculations of migration speed, we consequently overestimate the overall migration speed across the entire journey, as well as over the first migration leg (see Section 4).

**Table 1:** Summary statistics of general linear models of study year, birds’ sex, and age on migration speed in (a) autumn and (b) spring

	Estimate	*SE*	*t* value	*p*‐value
(a) A*utumn migration speed* (*df* = 33)				
Intercept	109.4	11.0	9.9	<0.01
Year_(2015)_	16.4	12.7	1.3	0.21
Sex_(male)_	−1.2	15.2	−0.1	0.94
Age_(2nd calendar year)_	3.8	9.4	0.4	0.69
(b) *Spring migration speed* (*df* = 22)				
Intercept	148.2	17.3	8.6	<0.01
Year_(2015)_	9.8	17.7	0.6	0.59
Sex_(male)_	−15.0	22.4	−0.7	0.51
Age_(2nd calendar year)_	5.6	13.9	0.4	0.69

Unfortunately, not all the recovered geolocators contained data of full annual schedules, and thus, our analyses are based on 41 individual tracks. Sample sizes for specific migration parts are as following: full autumn migration *n* = 36; autumn first leg *n* = 37; autumn second leg *n* = 38; full spring migration *n* = 26; spring first leg *n* = 28; spring second leg *n* = 26; full annual tracks *n* = 24.

We used nested ANOVA to test for differences in migration speed through different parts of the migration irrespective of the season. Two‐tailed paired t tests were used to compare migration speeds in autumn and spring of individual birds. Further, we used linear models to test whether there is a relationship between departure dates and migration speed of different parts of the migratory journey, as well as between arrival and departure dates.

## 3 RESULTS

### 3.1 Migration speed through different parts of the journey

**Figure 2:**
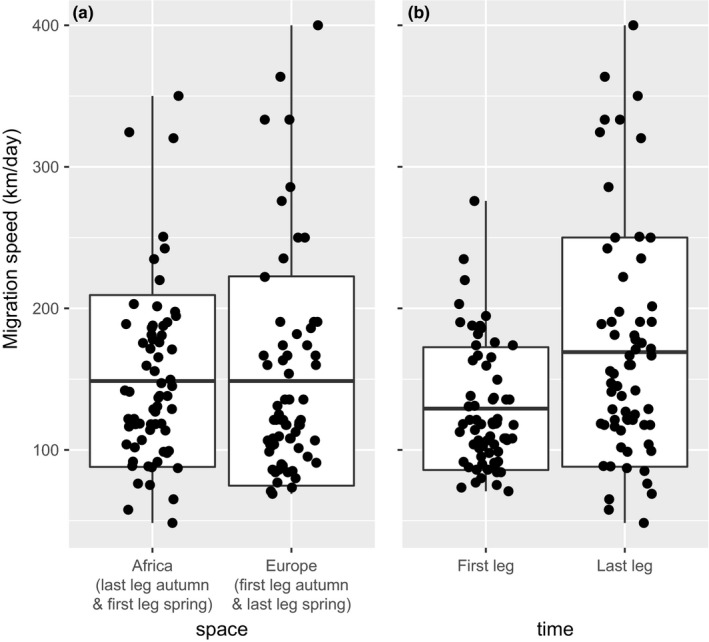
Comparison of migration speeds through different parts of the migration irrespective of the season. (a) Migration speeds across spatial scales, (b) migration speeds across temporal scales. Horizontal lines depict mean values, boxes—*SD*, vertical lines—range of data, and points denote individual data

We found no statistical differences in migration speed between the two spatial parts of the migration (*F*_1,89_ = 0, *p* = 1; Figure 2a) when pooling data of both seasons with speeds “through Africa” *sensu lato* (the second leg in spring & the first leg in spring) averaged at 148.7 ± 60.7 km/day (*SD*) and “through Europe” *sensu lato* (the first leg in autumn and second leg in spring) at 148.7 ± 73.9 km/day. On the contrary, we found the last leg to be significantly faster (*F*_1,89_ = 12.5, *p* < 0.001; Figure 2b), with migration speeds through the first migration leg 129.2 ± 43.4 km/day and through the last migration leg after the Sahara crossing 169.1 ± 81.0 km/day (data pooled for both seasons).

### 3.2 Migration speed in autumn and spring

**Figure 3:**
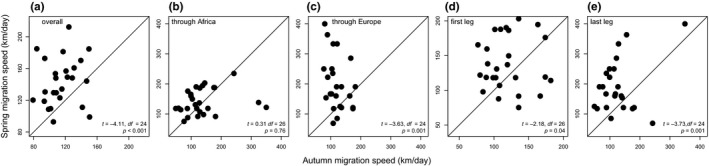
Comparison of individual migration speeds in autumn and spring, including speeds (a) over the entire migration, (b) through Africa, (c) through Europe, (d) the first leg of migration, and (e) the last leg of migration after the Sahara. Values below the diagonal line denote cases when the individual migrated faster in autumn that in spring (summary statistics of two‐tailed paired t tests are given within each panel)

**Figure 4:**
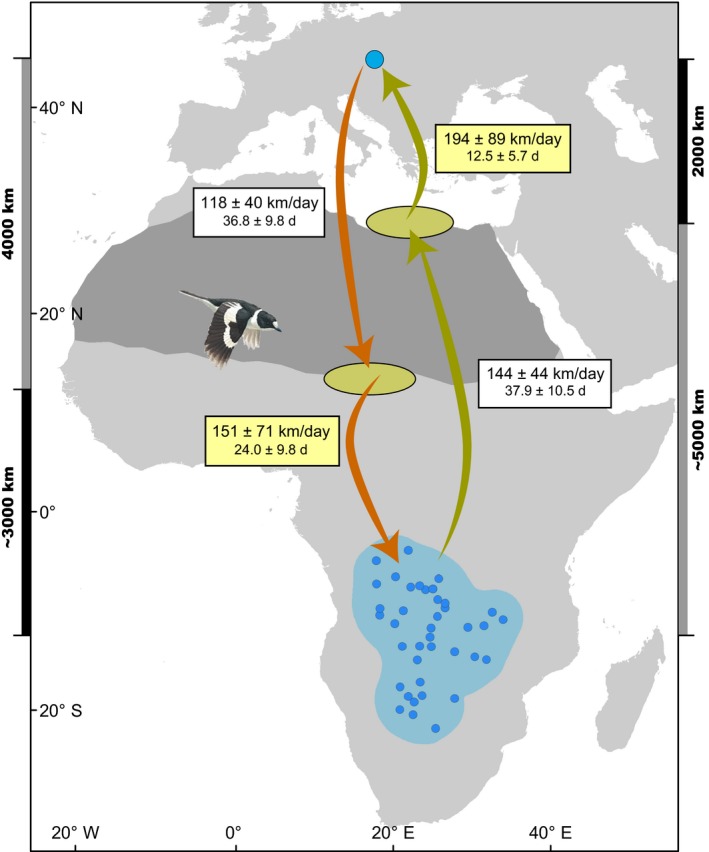
Average migration speed (km/day) and duration (days, d) ±*SD* of the tracked collared flycatchers over different legs of the migratory journey. Sprint migration over the last migration leg is highlighted in yellow. Larger blue circle denotes breeding site, light blue area—nonbreeding range with median coordinates of individual nonbreeding sites depicted as small blue circles. Arrows schematically illustrate different migration legs but do not depict precise migration routes taken. Map is shown in azimuthal equidistant projection centered at 0° latitude and 20°E longitude. The collared flycatcher illustration © Birds of Armenia Project

Average migration speed over the entire journey was significantly faster in spring (144.2 ± 30.4 km/day) compared to autumn (120.9 ± 26.1 km/day; Figure 3a). We found that for only four individuals (of 24) the average migration speed was lower in spring (Figure 3a), highlighting the overall faster spring migration on the individual level. We also found that birds increased their migration speed over the last leg of migration after the Sahara crossing (Figure 4) in both seasons. Spring migration speeds were typically faster for both migration legs (Figure 3d,e), with particular advancement through Europe (Figure 3c).

**Figure 5:**
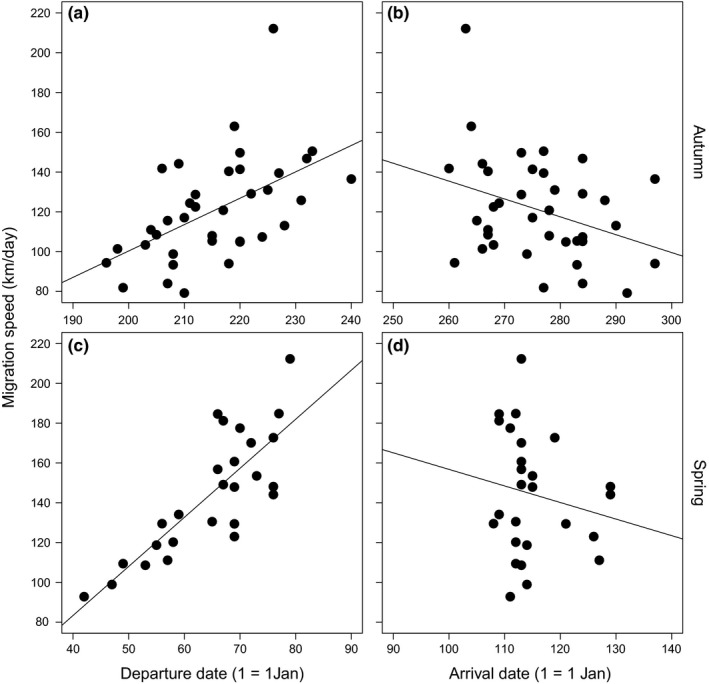
Ordinary least squares regression between overall migration speed and (a, c) migratory departure and (b, d) arrival dates in autumn and spring

We found a positive relationship between departure date and the overall migration speed in both seasons (autumn: *b* = 1.33 ± 0.35 (*SE*), *F*_1,35_ = 14.0, *p* < 0.001, *r* ^2^ = 0.29; spring: *b* = 2.47 ± 0.36, *F*_1,24_ = 45.9, *p* < 0.001, *r* ^2^ = 0.66; Figure 5a,c). This phenomenon was largely caused by faster migration of the late departing individuals through their first leg of migration (autumn: *b* = 1.45 ± 0.58, *F*_1,37_ = 6.3, *p* = 0.02, *r* ^2^ = 0.15; spring: *b* = 3.13 ± 0.63, *F*_1,26_ = 24.3, *p* < 0.001, *r* ^2^ = 0.48). The speed of the last migration leg was similar for all individuals, irrespective of their departure (autumn: *b* = 0.65 ± 1.15, *F*_1,35_ = 0.3, *p* = 0.57, *r* ^2^ = 0.01; spring: *b* = 1.71 ± 1.79, *F*_1,24_ = 0.9, *p* = 0.35, *r* ^2^ = 0.04).

**Figure 6:**
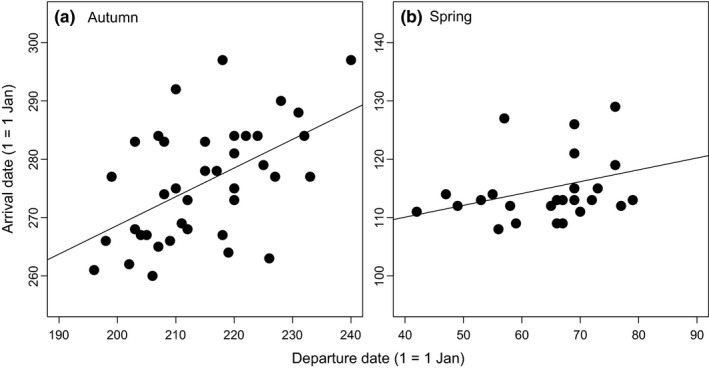
Ordinary least squares regression between migratory departure and arrival dates in (a) autumn and (b) spring

There was a negative relationship between arrival date and migration speed in autumn (*b* = −0.90 ± 0.42, *F*_1,35_ = 4.5, *p* = 0.04, *r* ^2^ = 0.11; Figure 5b), but not in spring (*b* = −0.83 ± 0.99, *F*_1,24_ = 0.7, *p* = 0.41, *r* ^2^ = 0.03; Figure 5d). However, when excluding one outlaying individual whose autumn migration speed was higher than 200 km/day, the relationship between autumn migration speed and nonbreeding arrival date was not significant (*b* = −0.57 ± 0.36, *F*_1,34_ = 2.5, *p* = 0.12, *r* ^2^ = 0.07). There was a significant and positive relationship between the departure and the arrival date in autumn (*b* = 0.49 ± 0.13, *F*_1,37_ = 14.3, *p* < 0.001, *r* ^2^ = 0.28; Figure 6a), but not in spring (*b* = 0.20 ± 0.12, *F*_1,24_ = 2.9, *p* = 0.10, *r* ^2^ = 0.11; Figure 6b). We found a weak positive relationship between autumn arrival date and migration speed of the last migration leg after the Sahara crossing (*b* = −0.04 ± 0.02, *F*_1,38_ = 3.6, *p* = 0.07, *r* ^2^ = 0.09), while there was no significant relationship between the speed through the first leg of the migration before Sahara and arrival time (*b* = −0.02 ± 0.04, *F*_1,37_ = 0.3, *p* = 0.56, *r* ^2^ = 0.01). Spring arrival date was not significantly related to migration speed of the last migration leg (*b* = 0.01 ± 0.01, *F*_1,24_ = 0.2, *p* = 0.63, *r* ^2^ = 0.01), nor with the first migration leg (*b* = 0.01 ± 0.02, *F*_1,24_ = 0.2, *p* = 0.88, *r* ^2^ < 0.01).

## 4 DISCUSSION

In this study, we first show that long‐distance migratory collared flycatchers adopt similar migration strategies in autumn and spring by increasing their migration speed in the last part of the journey after crossing the Sahara Desert. This supports the Alerstam's sprint migration hypothesis (Alerstam, 2006). Second, we reveal that migration through the last leg of the journey is faster in spring than in autumn, largely accounting for an overall faster spring migration and possibly indicating higher pressure for early arrival in spring. We also demonstrate that the overall migration speed in both seasons depends on departure date and late departing individuals are (at least partially) able to catch up with their early departing conspecifics to arrive at the destination at a similar time. Again, this phenomenon is more pronounced in spring, highlighting the possibly higher pressure for early arrival in this season. Our third hypothesis is partially supported—there is a positive trend for relationship between arrival date and migration speed through the last leg in autumn, but not in spring. It may be that in spring, most individuals exhaust themselves to the sustainable limits leaving little room for individual variation.

### 4.1 Sprint migration

As Alerstam (2006) coined the “sprint migration” hypothesis, he exemplified migration speeds of two male ospreys *Pandion haliaetus* at the tail end of their migrations in autumn and spring. None of the birds exhibited sprint migration in autumn, but results were ambiguous for one in spring. Likewise, GPS‐tracked honey buzzards *Pernis apivorus* and Montagu's harriers *Circus pygargus* did not show sprint migration to finish their journeys in either of the seasons (Vansteelant et al., 2015). Nilsson et al. (2013) also report no seasonal differences in the overall migration duration for thermal soaring migrants contrasting the findings in songbird migration. Ring recoveries have shown that songbirds tend to increase their migration speed along the route (Ellegren, 1993; Hedenström & Pettersson, 1987), providing indirect evidence for adoption of sprint migration strategy. Geolocator‐tracked swifts *Apus apus* showed no clear pattern of changes in travel rate between early and late stages of migration, while, similar to our tracked flycatchers, the swifts showed increased speed during the barrier crossing (Åkesson, Klaassen, Holmgren, Fox, & Hedenström, 2012). Rapid crossing of ecological barriers have also been demonstrated in other songbirds (Adamík et al., 2016; Ouwehand & Both, 2016) as well as waders (Klaassen, Alerstam, Carlsson, Fox, & Lindström, 2011; Pakanen et al., 2018). However, a comprehensive comparison of migration speeds in songbirds over different parts of their journey is still lacking due to former weight limitations of tracking devices.

The changes in speed over different legs of migration found in collared flycatchers may alternatively be explained by a reduction in migration speed over the first migration leg rather than advancement over the last leg. The overall slower speed during the first migration leg might arise due to prolonged stopovers for fuel accumulation before the cross‐desert flight or simply by weather‐induced delays of Sahara crossing. However, the latter should affect the migration speed both ways as some individuals may delay the Sahara crossing, while others may rush it to catch the optimal conditions. Furthermore, it has been recently shown that collared flycatchers routinely cross the Sahara Desert in flights reaching speeds of up to 1,000 km/day (Adamík et al., 2016; see Section 2). Therefore, we included the Sahara crossing in the first migration leg to diminish the effect of the lengthy pre‐desert stopovers on the overall speed of the first migration leg.

It is to be noted that our calculated migration speeds through the first leg are likely an overestimation due to the exclusion of predeparture fueling period (Alerstam, 2003). While accurate measure of individual predeparture fueling times is currently not possible using remote tracking techniques, Zhao et al. (2018) estimated that predeparture fueling in waders of various body sizes can take longer than the actual movement period between breeding and nonbreeding sites, leading to overestimation of migration speed by up to 60%. Unfortunately, we lack similar data on predeparture fueling durations in flycatchers before either autumn or spring migrations. In the Zhao et al. (2018) study, they also found a positive relationship between predeparture fueling duration and lean body mass of the species. Following their calculations, the predeparture fueling in collared flycatchers may range roughly between 5 and 10 days (depending on the on‐site specific intake rates). In such case, our migration speed for the first leg would be overestimated by 14%–25% in autumn and 13%–23% in spring. After the flight across the Sahara Desert and the completion of the first migration leg, flycatchers’ fuel reserves are presumably depleted and need to be renewed before continuing the migration. The last migration leg therefore may reflect nearly true migration speed including both fueling and flight to the final destination. Therefore, the difference in the observed migration speeds between the first and the last legs of migration is likely even larger with more pronounced sprint migration when considering the predeparture fueling as part of the first migration leg.

### 4.2 Seasonal differences in time‐selected migration

It is widely accepted that pressure for timely arrival in spring is higher due to the strong relationship between early arrival, breeding time, and subsequent reproductive success (Kokko, 1999; Wiggins, Pärt, & Gustafsson, 1994). We have also shown that there is such positive relationship between early arrival and early breeding in our study population (*b* = 1.26 ± 0.28, *F*_1,21_ = 20.73, *p* < 0.001, *r* ^2^ = 0.50; Briedis et al. 2018). Differences between spring and autumn migration durations are largely induced by reduction in stopover duration; the increase in flight speeds contributed relatively little (Nilsson et al., 2013; Schmaljohann, 2018). As the stopover durations are tightly linked to fueling rates (Schaub, Jenni, & Bairlein, 2008), it may be that the environmental conditions like food abundance and day length (i.e., the time available for foraging) at least partially explain the higher migration speeds observed in spring. Disentangling the intrinsic behavioral adaptations of individuals from those induced by changes in the environment is challenging and remains to be accomplished. However, at least in the case of collared flycatchers, the feeding conditions should be better during the autumn migration with higher food abundance along the route, while having similar daylight hours for feeding in both seasons (Bauchinger & Klaassen, 2005). Thus, we propose that competition for resources at the final destination rather than improved environmental conditions *en route* may better explain the faster overall spring migration as well the sprint finishes observed in the collared flycatchers.

Competition for resources at the nonbreeding sites (Lindström & Alerstam, 1992; Price, 1981) may also explain why autumn migration schedules of long‐distance migrants are tightly linked to the time of completion of breeding (Briedis, Hahn et al., 2018; Conklin, Battley, Potter, & Fox, 2010). Many long‐distance migrants likely begin the autumn migration as soon as their respective breeding cycles are over and they are in a physiological condition required for migration. With the progression of autumn, the Northern Hemisphere experiences depletion in resources for long‐distance migrants, while on the contrary, autumn marks the onset of rainy season in the Sahel where conditions are getting increasingly better. Thus, Afro‐Palearctic migrants would benefit from early and fast migration through Europe but, in case there is no competition for early arrival at the nonbreeding sites, could afford prolonged stopovers after the Sahara crossing as the prey abundance flourishes. Though, the observed sprint migration of collared flycatchers during autumn may suggest competition at the nonbreeding grounds. Long‐distance migrants spend several months at the nonbreeding sites where many species undergo full or partial feather molt and high‐quality molting sites may be the limited resource that birds compete for (Greenberg, 1986; Sherry & Holmes, 1996; Studds & Marra, 2005; Stutchbury, 1994).

The predeparture fueling (not considered in our calculations of the overall migration speed) should similarly affect the overall migration speed in autumn and spring given the fueling rates at breeding and nonbreeding destinations are similar. We found that spring migration was on average 11 ± 14 days (*SD*) shorter than autumn migration. If the observed pattern of faster spring migration originates solely by the amount of fat stored during the fueling, individual fat scores at departure in autumn would have to be much lower than at departure in spring. This would slow down the early parts of the migration as birds need to stop more often and/or for more extended periods to fuel for migratory flights. Even though this fits the pattern found in collared flycatchers where the migration speed through the first leg was slower in autumn compared to spring, it remains to be tested in the field by measuring fuel loads of birds upon migratory departure.

### 4.3 What determines arrival time?

Migration schedules of long‐distance migrants seem to be under tight endogenous control in autumn and spring alike (Berthold, 1991; Gwinner, 1996). In the closely related pied flycatcher *Ficedula hypoleuca*, spring arrival largely depends on the departure time from Africa, rather than migration speed (Ouwehand & Both, 2017). On the contrary, semicollared flycatcher *Ficedula semitorquata* spring arrival at breeding sites in Bulgaria differed significantly between years and was related to the environmental conditions *en route* and spring green‐up at the breeding site (Briedis, Hahn, & Adamík, 2017; Briedis, Träff et al., 2016). Thus, migration speed through the last leg of migration reflected the arrival time more closely than departure time in semicollared flycatchers. Our results show that both strategies, that is, early departure and slow migration, and late departure and fast migration, are used by the collared flycatchers possibly reflecting differences in fueling strategies for the early parts of migration.

None of our geolocator‐tracked birds adopted the strategy of early departure and fast migration speed. This may be due to physiological constraints and early departing individuals might recognize that they should depart earlier to arrive on time as they may be incapable of sustaining high migration speed throughout the journey. Unfavorable conditions *en route* or at the breeding sites may also be of high relevance in this regard (Briedis et al., 2017; Tøttrup et al., 2012). Migrating in spring before the environment is suitable implies high mortality risk. Late departing individuals might spend the extra time before the onset of migration to store additional fuel reserves for the early stages of migration. Such strategy would allow completing the first leg of migration without stopping‐over for extensive fueling. If this is the case, we may expect to see the pattern shown by the collared flycatchers when late departing individuals are those migrating faster through the first leg of the migration. Such pattern has also been found in several warbler species of genus *Sylvia* (Fransson, 1995) as well as in a number of other short‐ and long‐distance migrants (Ellegren, 1993) highlighting the importance of predeparture fueling as a determinant for migration speed through early stages.

Evidence suggests that in long‐distance migrants the onset of migration is less flexible (Doren, Liedvogel, & Helm, 2017; Gwinner, 1989), while the migratory journey itself has a great potential for flexible adjustments (Briedis et al., 2017; Marra, Francis, Mulvihill, & Moore, 2005; Tøttrup et al., 2012; van Wijk et al., 2012). Within the Afro‐Palearctic bird migration system, cues about the environmental conditions at the destination can be obtained only after crossing the Sahara Desert (and possibly Mediterranean Sea). Such circumstances present a situation when speed through the first leg of migration may offer limited adjustments in accordance with the environmental conditions at the breeding grounds. Acceleration of migration speed through the last leg of the journey may play an important role not only in intraspecific competition but also in adjusting migration timing in accordance with phenology of each particular spring as well as for climate change (Schmaljohann & Both, 2017).

## CONFLICT OF INTEREST

None declared.

## AUTHOR CONTRIBUTIONS

MB conceived the idea and study design. SH provided geolocators. MB, MK, and PA collected data. PA organized and coordinated work in the field. MB with help of SH analyzed the data. MB wrote the manuscript with substantial contributions from all other authors.

## DATA ACCESSIBILITY

Raw sun events of geolocator data: Dryad: https://doi.org/10.5061/dryad.v51p331.
